# Association of Gender and Personal Choices with Salaries of New Emergency Medicine Graduates

**DOI:** 10.5811/westjem.33606

**Published:** 2024-09-09

**Authors:** Fiona E. Gallahue, Louis J. Ling, Leo Quigley, Dian Dowling Evans, Edward Salsberg, Robert E. Suter, Catherine A. Marco

**Affiliations:** * The University of Washington, Department of Emergency Medicine, Seattle, Washington; † University of Minnesota, Department of Emergency Medicine, Minneapolis, Minnesota; ‡ George Washington University Fitzhugh Mullan Institute for Health Workforce Equity, Washington, DC; § Emory University Nell Hodgson Woodruff School of Nursing, Atlanta, Georgia; ∥ Sam Houston State University College of Osteopathic Medicine, Office of the Dean, Conroe, Texas; ¶ Uniformed Services University of the Health Sciences, Department of Military Medicine, Bethesda, Maryland; # Penn State Health Milton S. Hershey Medical Center, Department of Emergency Medicine, Hershey, Pennsylvania

## Abstract

**Objective:**

The medical literature has demonstrated disparities and variability in physician salaries and, specifically, emergency physician (EP) salaries. We sought to investigate individual physician characteristics, including sex and educational background, together with individual preferences of graduating EPs, and their association with the salary of their first job.

**Methods:**

The American College of Emergency Physicians and the George Washington University Mullan Institute surveyed 2019 graduating EPs. The survey included respondents’ demographic and educational background, post-training job characteristics and location, hospital characteristics, importance of different personal priorities, and starting salaries. We performed a multivariable regression analysis to determine how salaries were associated with job types and individuals’ characteristics.

**Results:**

We sent surveys to 2,192 graduating residents in 2019. Of these, 487 (22.2%) responded, and 270 (55.4%) accepted first-time clinical jobs and included salary data (12.3% of all surveys sent). Male sex, osteopathic training, and full-time work were significantly associated with higher salary. Men and women prioritized different factors in their job search. Women were more likely to consider such factors as parental leave policy, proximity to family, desired practice setting, type of hospital, and desired location as important. Salary/compensation was considered very important by 51.8% of men and 29.6% of women. Men’s median salary was $30,000 more than women’s (p = 0.01, 95% CI +$6,929 −+$53,071), a significant pay differential.

**Conclusion:**

Salaries of graduating emergency medicine residents are associated with the resident’s sex and degree type: doctor of osteopathic medicine or doctor of allopathic medicine. Multiple factors may contribute to men having higher salaries than women, and some of this difference reflects different priorities in their job search. Women were more likely to consider job conditions and setting to be more important, while men considered salary and compensation more important.

Population Health Research Capsule What do we already know about this issue?

*Physician salaries have demonstrated disparities and variability. Multiple factors may be associated with salary differences.*

What was the research question?

*What factors impact salaries of emergency medicine graduates?*

What was the major finding of the study?

*Men’s median salary was $30,000 more than women’s (p = 0.01, 95% CI +$6,929 − +$53,071).*

How does this improve population health?

*This study suggests that the salaries of new EM graduates are impacted by multiple factors including gender.*

## INTRODUCTION

### Background

Previous reports have demonstrated disparities and variability in physician salaries and, specifically, emergency physician (EP) salaries by a variety of factors.^1^ ^–^ ^7^ Disparities by sex with men earning more than women is well documented,^3^ ^,^ ^5^ ^–^ ^7^ but the possible causes of this disparity along with the association of other factors with salary differences is not as well described. We review possible associations with this variability in starting salaries of new EP residency graduates in 2019.

### Importance

Graduating emergency medicine resident physicians have personal characteristics that may influence the selection of their very first job. These include personal preferences that influence their job-selection decisions and may impact the final salary of the job they ultimately select. Individuals may have preferences in selecting their job. The choices made by women and men may be different. Unfortunately, very little is known about these factors to guide young EPs as they go through the process of job selection.

### Goals of This Investigation

We present the results of an analysis of a survey of EPs completing their residency training that included a wide range of questions about the type of jobs they considered, their personal and professional demographics, factors that impacted their job selection decision and, finally, the job that was chosen and resulting salary. Our goal was to describe the association of personal traits and job characteristics with the salary of the first job, including the impact of the graduate’s sex. We compared and contrasted the priorities of women and men in their job hunt. We performed a secondary regression analysis to investigate the impact of sex alone on salary, independent of these preferences. By focusing on individuals obtaining their very first job post training, we eliminated the impact of mid-career job changes or job changes for career advancement in assessing the impact of such factors as the EP’s sex.

## METHODS

### Survey Instrument

As part of a larger initiative to study the workforce needs for EPs in the near future, the American College of Emergency Physicians (ACEP) and the George Washington University Mullan Institute fielded a survey to all 2019 graduating EM residents, approximately 4–8 weeks prior to graduation.^8^ This study reviewed a subset of graduating EM residents’ personal characteristics, educational background, and characteristics of their job choices. We used a multivariable quantile regression analysis to estimate the possible impact of some of these factors on the salaries of their first post-residency clinical job.

The research design and survey were approved by the George Washington University Institutional Review Board. We adapted the survey tool from similar surveys used by the Mullan Institute for studies of other physician specialty graduates and further adjusted it based on feedback from subject experts and pilot-testing among EPs. The survey questions offered a four-point Likert scale (very important, important, of little importance, not important at all), with a not-applicable option, for priority factors in a job. (See Supplementary Material.)

### Survey Dissemination

The survey was distributed via email in May and June 2019, approximately 4–8 weeks prior to their graduation, to 2,192 residents within the ACEP database who reported they would complete their training program in 2019. The 2,192 residents represented approximately 97.3% of the 2,253 residents in their final year of training on December 31, 2018, as reported by the American Board of Emergency Medicine.^9^ An original email was followed by up to three reminders for non-respondents. As an incentive, 10 gift cards with a value of $150 were distributed randomly to respondents. The online survey used REDCap (Research Electronic Data Capture) software, hosted at George Washington University Mullan Institute.^10^

### Outcome and Measures

We collected data including respondents’ demographic and educational background, educational debt, post-training jobs, job market experiences, and factors influencing post-training job plans. We looked at these factors and their association with the salaries of the first job selected by the 270 graduates of EM residencies who had accepted a clinical position post-residency and supplied their base salary.

### Statistical Analysis

Physician income is associated with factors such as specialty, sex, race, ethnicity, academic practice, board-certification status, and work intensity.^2^ ^–^ ^6^ The primary statistical analysis involved an overview of these factors.^11^ In a secondary analysis we used quantile regression in a multivariable analysis estimating the remaining salary differences by sex when multiple factors were controlled for. Selection of variables for the model was guided by a similar regression analysis in a survey of all US-based ACEP members in which the variables used were chosen to represent income-associated factors including specialty, sex, race, ethnicity, academic practice, board-certification status, and work intensity.^1^ ^–^ ^7^ The variables included in the model were as follows: age; working full vs part time; racial minority status (Black, American Indian or Alaskan Native vs White or Asian); ethnicity (Hispanic vs non-Hispanic); urban vs rural or semi-rural practice (where “urban” means being located in a city of 50,000 or more residents, “semi-rural” refers to “urban clusters” of 2,500 to 49,999 residents as defined for the 2010 census, and “rural” refers to areas with fewer than 2,500 residents); employment in an academic medical center; country of medical school (international medical graduate vs graduate of US and Canadian medical school); medical degree type (doctor of allopathic medicine vs doctor of osteopathic medicine [MD vs DO]); whether working a secondary job; and working for a for-profit vs a non-profit organization.

All respondents were asked to self-define their situation according to the stated criteria. For the regression model we treated the categorical income variable as a point variable by defining annual income as the mid-point of the $10,000 ranges used in the survey questionaire, while age was treated as a continuous (whole number of years) variable. All other variables were dichotomous with values derived from yes/no survey responses, with the exception of whether working for for-profit or non-profit organizations where the response options were yes/no/don’t know. To avert the loss of 51 respondents who stated that they did not know the for-profit or non-profit status of their employing organization, the value of this variable for “don’t know” respondents was imputed as the mean of the yes/no response value across respondents who did respond yes or no; it was assumed that not knowing for-profit or non-profit organizational status was randomly distributed across both sexes.

Quantile regression is typically used when the assumptions of normal distribution do not hold, as is often the case in salary distributions.^12^ This method enabled us to generate results at the lower (25%) and higher (75%) income quartiles of the income distribution as well as at the distribution’s center (median). The results of the lower and higher quantile regression analysis are not separately reported as they closely resembled the result at the median. They are available from the authors.

Owing to missing data among the 270 respondents, the final regression analysis included 258 observations. The 13 independent variables used in the model and 258 observations allowed the regression model to adhere to the general overfitting guideline of approximately 20 observations per outcome variable.^11^ We carried out a test for overfitting out using the Stata “overfit” command, which reports both in-sample and out-of-sample shrinkage statistics, for each regression result.^13^ Data were analyzed using Stata 16^14^ (StataCorp, LLC, College Station, TX).

## RESULTS

A total of 487 graduating residents returned the survey. Of these, the 270 respondents who accepted first-time clinical jobs and included salary data comprised the group that was analyzed. Three of the 270 respondents who did not identify a binary gender were excluded from the sex-specific analysis. Of the 270 respondents, 258 had complete data that could be analyzed for the regression. Of the 270, 169 (62.6%) self-identified as men, 98 (36.3%) as women, and three (1.1%) preferred to self-describe or not answer. Of 262 who gave their age, 234 (89.3%) were under 36 years old. Of 268 respondents, 211 (78.7%) were White, 35 (13.1%) were Asian, five (1.9%) were Black, and 17 (6.4%) were “other” or more than one race. Of the 270 graduates, 171 (63.3%) had three years of training, 94 (34.8%) had four years and five (1.9%) had ≥5 years.

Compared to ABEM data, the proportion of respondents who reported completing three-year programs was less than the percentage of those completing three-year programs nationally at the end of calendar year 2018 (63.6% vs 70.2%) and skewed more White than EM residents nationally (78.7% vs 63%), and less Black (1.9% vs 4%) than residents in training during 2018–2019.^9^ Of 264 respondents with data, 93 (35.2%) had educational debt exceeding $300,000, while the median educational debt was $237,500. Almost three quarters (72.4%) reported “quite a bit” or “a great deal” of salary variability in their job search (Table 1).

**Table 1.** Perceived variability of physicians’ salaries.^*^

How much salary variability (in terms of total annual income) was there?	Number	Percent
Quite a bit	138	51.5%
A great deal	56	20.9%
Very little or none	51	19.0%
Only got one offer so can’t say	23	8.6%
Total	268	100

* 268 of the 270 responded to this question.

### Multivariable Analysis of Salary Differences

To assess the multiple factors that appeared linked to the salaries earned by newly graduating residents we used a quantile regression analysis to control for many of these factors together at different quantiles of the income distribution. The assessment accounted for both part-time and full-time work. The most statistically significant factor was gender gap, with men making a median $30,000 more than women ($244,200 vs $214,200), followed by DO graduates who made a median $30,000 more than their MD counterparts (median $274,200 for men vs $244,200 for women who are DO graduates), and full-time workers making a median of $43,000 more than those working part-time ($287,349 for men vs $257,349 for women who are full-time workers) (Table 3).

Other factors that were not significant but trended toward a positive impact on salary were non-academic center employment compared to academic and semi-rural/rural compared to urban jobs. Two thirds of graduates accepted jobs at sites in cities despite lower salaries. Men were more likely to accept jobs associated with higher pay in smaller communities, non-academic settings, and smaller hospitals.

Sensitivity analysis included running the model with and without the for-profit variable. With and without imputation of the values of the for-profit variable did not significantly alter the results, which appeared to be robust to minor variations in the model. Similarly, the results did not change substantially between linear regression and quantile regression, likely because the income distribution in this sample appeared to closely follow a normal distribution based on Shapiro-Wilk and Shapiro-Francia tests of normality and Stata’s “sktest” for skewness and kurtosis.

### Factors Considered by Women and Men in Their Job Search

Respondents rated factors they considered in selecting a job with response options ranging from “very important” to “not important at all.” We focused on factors rated as “very important” compared to other responses (excluding “not applicable”), reported in Table 2A, and “very important” and “important” together compared to of “little importance” and “not important,” reported in Table 2B. The rationale behind this is that “very important” factors could be considered critical in the job selection decision and their absence could eliminate a possible job, while “important” factors could be considered nice to have and, therefore, only a swing factor in job selection if the “very important” criteria were met. The factors are listed in order of the percent difference of preferences of women over men. Factors at the top of Tables 2A and 2B have the greatest difference in what women considered important compared to men; factors at the bottom are those that men considered more important than women.

**Table 2A.** “Very important” factors influencing job selection for men and women^*^ sorted by difference in percent.^*^

	“Very important” for women			“Very important” for men					
Factor is “very important”	Freq. women	N^**^	Percent women	Freq.men	N^**^	Percent men	Diff in percent	Pearson chi sq	*P*-value
Parental leave policy	20	79	25.3	6	142	4.2	21.1	21.7520	<0.001^*^
Proximity to family	41	93	44.1	42	164	25.6	18.5	9.2653	0.002^*^
Jobs/practice in desired practice setting	52	98	53.1	68	168	40.5	12.6	3.9591	0.05^*^
Type of hospital	22	98	22.4	16	164	9.8	12.6	7.9700	0.01^*^
Jobs/practice in desired location	76	98	77.6	110	167	65.9	11.7	4.03	0.05^*^
Employment for spouse/partner	26	75	34.7	33	142	23.2	11.5	3.2373	0.07
Patient population to be served	24	97	24.7	22	162	13.6	11.1	5.1753	0.02^*^
Weather	25	96	26.0	31	166	18.7	7.3	1.9643	0.16
Availability of mentors	16	93	17.2	17	163	10.4	6.8	2.4204	0.12
Supportive academic environment	13	92	14.1	12	160	7.5	6.6	2.8736	0.09
Availability of part-time position	10	76	13.2	11	137	8	5.2	1.4468	0.23
Type of community (eg, rural/urban)	21	97	21.6	27	165	16.4	5.2	1.1406	0.29
Job/practice meets visa requirements	4	26	15.4	6	54	11.	4.4	0.2930	0.59
Frequency of weekend duties	23	98	23.5	32	167	19.2	4.3	0.6968	0.40
Frequency of overnight shifts	27	98	27.6	39	165	23.6	4.0	0.5012	0.48
Opportunities for teaching	11	94	11.7	15	162	9.3	2.4	0.3890	0.53
Staying in the same city/region as EM training	8	89	9.0	11	154	7.1	2.1	0.2666	0.61
Use of NP, PA, and other clinical staff	3	93	3.2	4	161	2.5	0.7	0.1209	0.73
Length of work day	43	98	43.9	73	168	43.5	0.4	0.0045	0.95
Cost of living	14	97	14.4	24	164	14.6	−0.2	0.0020	0.96
Opportunities for research	0	90	0	2	160	1.2	−1.2	1.1341	0.29
Predictable work day start and end times	33	97	34	67	167	40.1	−6.1	0.9700	0.33
Organizational structure of practice	14	97	14.4	37	164	22.6	−7.6	2.5611	0.11
Partnership opportunity	6	82	7.3	23	149	15.4	−8.1	3.1760	0.08
Other factors	2	27	7.4	7	47	14.9	−14.5	0.8996	0.34
Salary/compensation	29	98	29.6	87	168	51.8	−22.2	12.3975	<0.001^*^

Sorted in order of the difference in percentage of women responding as “very important” compared to men (column 7).

* *P* < .05 significance.

** Since not every respondent answered every question, N is the total number of women and men responding to the question.

*EM*, emergency medicine; *NP*, nurse practitioner; *PA*, physician assistant.

**Table 2B.** “Very important” and “important” vs of “little importance” and “not important at all”: factors influencing job selection for men and women sorted by difference in percent.^*^

	“Important” for women			“Important” for men					
Factor is “very important”	Frequency women	N^**^	Percent women	Frequency men	N^**^	Percent men	Difference in percent	Pearson chi square	*P*-value
Parental leave policy	51	79	64.6%	31	142	21.8	42.8%	39.7066	<0.001^*^
Job/practice meets visa requirements	9	26	34.6%	7	54	13%	21.6%	5.1425	0.02^*^
Patient population to be served	72	97	74.2%	91	162	56.2%	18.0%	8.4776	0.004^*^
Employment for spouse/partner	53	75	70.7%	75	142	52.8	17.9%	6.4636	0.01^*^
Availability of part-time position	32	76	42.1%	34	137	24.8%	17.3%	6.8317	0.01^*^
Staying in same city/region as EM training	30	89	33.7%	26	154	16.9%	16.8%	9.0029	0.003^*^
Availability of mentors	61	93	65.6%	82	163	50.3%	15.3%	5.6106	0.02^*^
Supportive academic environment	41	92	44.6%	50	160	31.2%	13.4%	4.4889	0.03^*^
Rural vs urban	71	97	73.2%	100	165	60.6%	12.6%	4.2713	0.04^*^
Frequency of weekend duties	69	98	70.4%	98	167	58.7%	11.7%	3.6434	0.06
Type of hospital	70	98	71.4%	98	164	59.8%	11.6%	3.6330	0.06
Proximity to family	68	93	73.1%	102	164	62.2%	10.9%	3.1622	0.08
Opportunities for teaching	38	94	40.4%	53	162	32.7%	7.7%	1.5431	0.21
Frequency of overnight shifts	73	98	74.5%	117	165	70.5%	4.5%	0.3931	0.53
Opportunities for research	7	90	7.8%	6	160	3.75%	4.05%	1.8956	0.17
Organizational structure of practice	59	97	60.8%	94	164	57.3%	3.5%	0.3092	0.58
Use of NP, PA, and other clinical staff	29	93	31.2%	45	161	28%	3.2%	0.2983	0.59
Weather	59	96	61.5%	99	166	59.6%	1.9%	0.0841	0.77
Jobs in desired practice setting	86	98	87.8%	146	168	86.9%	0.9%	0.0401	0.84
Cost of living	63	97	64.9%	105	164	64%	0.9%	0.0227	0.88
Partnership opportunity	37	82	45.1%	66	149	44.3%	0.8%	0,0146	0.90
Jobs/Practice in desired location	94	98	95.9%	159	167	95.2%	0.7%	0.0718	0.79
Length of work day	90	98	91.8%	153	168	91.1%	0.7%	0.0459	0.83
Other factors	9	27	33.3%	17	47	36.2%	−2.9%	0.0606	0.81
Predictable work day start and end times	75	97	77.3%	137	167	82%	−4.7	0.8629	0.35
Salary/compensation	87	98	88.8%	162	168	96.4%	−7.6%	6.0595	0.01^*^

Sorted in order of the difference in percentage of women responding as “very important or important” compared to men (column 7).

* *P* < .05 significance.

** Since not every respondent answered every question, N is the total number of women and men responding to the question.

*EM*, emergency medicine; *NP*, nurse practitioner; *PA*, physician assistant.

**Table 3.** Quantile regression results (median).

Category	Characteristic	Variable	Number (percent)	Mean income difference ($)^1^	(95% confidence interval)	*P* > |t|	Median salary (male) ($) ^*^	Median salary (female) ($) ^*^
Demographics	Sex	Men	162 (62.8%)	$+30,000	(+6,929 −+ 53,071)	0.01^*^	244,200	214,200
		Women	96 (37.2%)	reference				
	Race^2^	Under-represented	3 (1.2%)	$+3,114	(−98,750 −+ 104,977)	0.95	247,313	217,313
		Non-minority	255 (98.8%)	reference				
	Ethnicity	Hispanic	12 (4.7%)	$−2,491	(−54,791 −+ 49,809)	0.93	241,708	211,708
		Non-Hispanic	246 (95.3%)	reference				
	Age	Increasing age	Mean	$−1,246	(−4,535 −+ 2,044)	0.46	242,954	212,954
Training	Training location^3^	International medical graduate	10 (3.9%)	$+48,096	(−9,067 −+ 105,529)	0.10	292,296	262,296
		US or Canadian graduate	248 (96.1%)	reference				
	Degree type^4^	DO degree	72 (27.9%)	$+30,000	(+5,133 −+ 54,867)	0.02^*^	274,200	244,200
		MD degree	186 (72.1%)	reference				
Job Characteristics	Work Setting	Academic	68 (26.4%)	$−24,539	(−50,226 −+ 1,507)	0.07	219,840	189,840
		Non-academic	190 (73.6%)	reference				
	Organization type	For-profit	91 (43.5%)	$−1,246	(−26,631 −+ 24,141)	0.92	242,954	212,954
		Nonprofit	118 (56.5%)	reference				
	Location	rural location<50,000	27 (10.5%)	$+35,605	(−475 −+ 71,685)	0.05	279,805	249,805
		>50,000 in city	231 (89.5%)	reference				
	Primary job	Full time	238 (92.2%)	$+43,149	(−1349 −+ 84,950)	0.04^*^	287,349	257,349
		Part time	20 (7.8%)	reference				
	Secondary Job	Has secondary job	70 (27.1%)	$−6,263	(−30,951 −+ 18,424)	0.62	237,936	207,936
		No secondary job	188 (72.9%)	reference				

1 Base salary only.

2 Under-represented minority of Black, Native American, and Alaskan compared to all others.

3 International medical graduate compared to Canadian and American schools accredited by the Liaison Committee on Medical Education and American Osteopathic Association.

4 *DO*, osteopathic physicians, compared to *MD*, allopathic physicians.

* *P* > |t|: *P*-value in regression table: *P* < .05 significance.

** This is the median salary for males/females with all other variables set to their null value, including the continuous variable for age.

† This table shows results for 258 respondents.

Generally, women considered lifestyle and work-life balance factors in their job-seeking choices more than men. Parental leave policy, proximity to family, the practice setting, the type of hospital, and location of the hospital were statistically more often cited by women as “very important” factors, while salary/compensation was statistically more frequently noted for men. When adding “nice to have” “important” factors to the “very important” factors, a different list of priorities was statistically different for women and men. For women, the parental leave policy, meeting visa requirements, the patient population, employment options for the spouse, staying in the same region as the training program, availability of mentors, supportive academic environment, and urban vs rural setting could be considered “nice to have”. Salary/compensation was the only factor statistically more important for men than for women.

## DISCUSSION

There are many factors associated with the salaries that graduates receive.^1^ ^–^ ^6^ Three factors are statistically significant. The most significant factor resulting in greater pay is the gender gap, followed by a DO degree and, not surprising, that full-time employment pays more than part-time employment.^15^ In comparison, a similar recent survey of all ACEP members found sex was significantly associated with base salary but type of degree was not.^1^ New osteopathic graduates are reported in other studies to be more inclined to choose positions in non-academic settings in smaller communities, both of which appear associated with higher salaries.^16^ ^,^ ^17^ A predominance of all graduates accepted jobs at sites in cities despite the lower salaries. This is not surprising when considering the “very important” preferences that are summarized in Table 2A.

There are annual surveys of employers that collect data on average salary and compensation by specialty such as the Modern Healthcare Physician Compensation Database and the Medical Group Management Association.^18^ ^,^ ^19^ Most surveys include all jobs for new and experienced physcians in different phases of their careers and usually do not differentiate between type of hospital, hospital site, and other factors that are inherent to their local community. The jobs may have different proportions of clinical and administrative or leadership responsibilities, which may make it difficut to compare between jobs. This ACEP-Mullen Institute survey is unique in that it focused specifically on starting salaries of graduates taking their very first entry-level job. Even for this well-defined type of position, there was “quite a bit” or “a great deal” of variance in the jobs they were offered, (Table 1) as noted by graduates.

A persistent gender gap has been identified in numerous studies, despite many years of attention in many professions, many other specialties; EM appears to be no different.^5^ ^,^ ^6^ ^,^ ^7^ ^,^ ^15^ ^,^ ^20^ ^,^ ^21^ ^,^ ^22^ We found a gap attributable to sex alone of $30,000 at the median, which was consistent across the income distribution. Reisdorff et al found a significant gender gap of $43,565 for a sample of all ACEP members, and Madsen et al found a smaller but still significant difference of $19,418 for academic EM faculty.^1^ ^,^ ^22^ The impact of sex in physician pay seems persistent throughout a career and could translate to an over $2 million net income difference over a 40-year career.^23^ Our results on difference in job preferences by sex suggest that the reasons for the gap may not be entirely systemic but may be at least partly associated with the different locations and type of jobs that men and women select.

Graduating residents have many factors to consider and each individual will have different personal priorities as well as different career goals. Women are statistically less likely than men to consider high salary as very important and more likely to consider more non-salary factors, as listed in Tables 2A and 2B, as more important than men. The long-term financial impact of these decisions are unknown and were not considered in the analysis, but these personal priorities may contribute to the unexplained and persistent salary differences by sex.

We confirmed that salary may be impacted, but less so, based on location of practice, size of the city and the setting, with academic and large hospitals paying less than smaller, rural and community hospitals. Men were more likely to accept jobs associated with higher pay in smaller communities, non-academic settings, smaller hospitals, or exclusively EM practice consistent with at least one other study.^24^ As an anticipated surplus of EPs is projected, its impact will be felt most directly by graduating residents who are new to the job market and most closely invested in the supply and demand.^25^ Of note, this survey was completed prior to the COVID-19 pandemic.

## LIMITATIONS

There were several limitations to the study. The sample of 270 residents analyzed and the 258 who had complete (or imputed) data for the regression analysis represents approximately 12% of all graduating residents in 2019; thus, while it cannot be considered a representative sample, it does provide insight into some of the factors considered by graduating residents that may be associated with their salaries. Understanding salary was not the primary purpose of the larger initiative, but the resulting dataset included salary data for respondents who had accepted a post-residency job. The analysis compared respondents’ base salaries without consideration of additional income sources such as incentive payments and may not represent all anticipated income. Like all surveys relying on self-reported data, the potential exists for inaccuracies or recall bias. Survey respondents were asked to report their salaries within $10,000 ranges; so, the point values used in the analysis presume that actual salaries were evenly distributed within each salary range, which could have resulted in some inaccuracy in salary reporting.

Although there is variability in supply and demand of emergency physicians by state, our intent was a national scope; thus, analysis of the salaries and job opportunities within specific regions or states was not performed. While the original survey tool has been used widely with graduates of other specialties, validation of this survey tool was limited to content-expert comments, pilot-testing within EM, and modification based on feedback received.^1^ We did not explore any numerical measures of factor loading or survey consistency.

The regression model was limited to 13 variables to address overfitting, necessitating difficult choices as to which variables to include and how best to construct dummy variables from categorical ones. Nevertheless, the results of a sensitivity analysis that found only minor changes in the results when alternative variable listings were explored suggest that the regression model was not sensitive to changes in model specification. Finally, the regression analysis used a sample that was slightly smaller than the main sample (258 vs 270) owing to missing data on some variables. It is assumed that the 258 were closely representative of the larger sample, given the small difference in numbers between the two samples.

## CONCLUSION

A sample of 2019 graduating EM residents reported variance in salaries that depended on several variables. Men and osteopathic physicians were paid significantly more in their first jobs than women and allopathic physicians. In exploring possible reasons for the gap in pay between men and women, women are statistically more likely than men to consider lifestyle factors such as parental leave policy, proximity to family, job location, practice setting, and type of hospital as priorities. Men are statistically more likely to consider salary and compensation more important than women. Nevertheless, the gap in pay between men and women EM graduates is not fully explained by the factors we were able to include in our analysis, and other explanations must be sought for the portion of the gender gap that remains unexplained.

## Supplementary Information



