# Supplementary material for: Association of Gender and Personal Choices with Salaries of New Emergency Medicine Graduates
Source: West J Emerg Med. 2024 Sep 9;25(5):800–8. doi: 10.5811/westjem.33606 (PMC11418865; doi:10.5811/westjem.33606)
Supplement: Supplementary file 1 [file wjem-25-800-s001.pdf]

Emergency Medicine Residents Survey (2019)

Data Dictionary Codebook

05/07/2019 5:11pm

#	Variable / Field Name	Field Label *Field Note*	Field Attributes (Field Type, Validation, Choices, Calculations, etc.)
Instrument: **Emergency Medicine Residents Survey** (emergency_medicine_residents_survey)			
1	record_id	Record ID	text
2	consent_description	Thank you for your interest in the Emergency Medicine Resident Survey, part of a research study directed by Edward Salsberg of the George Washington University Health Workforce Institute in collaboration with the American College of Emergency Physicians. Please review this important information about your rights and protections to decide if you wish to participate in the study.<br><br>Participation is Voluntary. Taking part in this research is entirely voluntary, and you may choose not to participate or to stop participating in the study at any time.<br><br>Study Purpose. The purpose of this study is to understand trends in the Emergency Medicine workforce and job market. Your answers will be analyzed for scholarly research and for reports to inform the emergency medicine community including training programs.<br><br>Time Required. This survey takes between 5 and 10 minutes to complete. You may skip any questions you do not want to answer, and you may stop your participation in this study at any time.<br><br>Potential Risks of Participation. Privacy and confidentiality are usually participants' most important concerns, and are discussed in detail below.	descriptive

		Privacy and Confidentiality Protections. All identifying information (e.g., email and IP addresses) will be separated from your responses immediately after you complete the survey. Your responses will be stored on secure research computer servers and data files will be available to authorized members of the research team except as required by law. All reports and published papers derived from the survey will only report aggregated results.<br><br>Benefits of Participation. Your participation will provide important information about the Emergency Medicine workforce and job market that will help the emergency medicine community.<br><br>Incentives. Award gift certificates of $150 each will go to ten randomly selected survey respondents. All survey respondents will be entered into the drawing for this award, which will be held immediately after the survey closes.<br><br>Study Approval and Further Information. This study has been reviewed and approved by the George Washington University Office of Human Research. Please contact Edward Salsberg, Principal Investigator (esalsberg@gwu.edu; 202-994-2049) or the George Washington University Office of Human Research (202-994-2715) for further information about your rights as a research participant.	
3	consent	ELECTRONIC CONSENT: Please select your choice below.<br><br>Clicking on the "Agree" button below indicates that:<br>• You have read the above information<br>• You voluntarily agree to participate<br>• You are at least 18 years of age	radio, Required<br>1 Agree<br>2 Disagree<br><br>Custom alignment: LV<br>Stop actions on 2
4	em_residncy	Section Header: *YOUR EMERGENCY MEDICINE TRAINING*<br>Are you completing an Emergency Medicine residency or fellowship in 2019?	radio<br>1 Yes, residency program<br>2 Yes, fellowship program<br>3 No

			Stop actions on 3
5	residency_state<br><br>Show the field ONLY if: [em_residncy] = '1'	Which state is your residency program located in?	dropdown<br>1 AL : Alabama<br>2 AK : Alaska<br>3 AZ : Arizona<br>4 AR : Arkansas<br>5 CA : California<br>6 CO : Colorado<br>7 CT : Connecticut<br>8 DE : Delaware<br>9 DC : District of Colombia<br>10 FL : Florida<br>11 GA : Georgia<br>12 HI : Hawaii<br>13 ID : Idaho<br>14 IL : Illinois<br>15 IN : Indiana<br>16 IA : Iowa<br>17 KS : Kansas<br>18 KY : Kentucky<br>19 LA : Louisiana<br>20 ME : Maine<br>21 MD : Maryland<br>22 MA : Massachusetts<br>23 MI : Michigan<br>24 MN : Minnesota<br>25 MS : Mississippi<br>26 MO : Missouri<br>27 MT : Montana<br>28 NE : Nebraska<br>29 NV : Nevada<br>30 NH : New Hampshire<br>31 NJ : New Jersey<br>32 NM : New Mexico<br>33 NY : New York<br>34 NC : North Carolina

			35 ND : North Dakota<br>36 OH : Ohio<br>37 OK : Oklahoma<br>38 OR : Oregon<br>39 PA : Pennsylvania<br>40 PR : Puerto Rico<br>41 RI : Rhode Island<br>42 SC : South Carolina<br>43 SD : South Dakota<br>44 TN : Tennessee<br>45 TX : Texas<br>46 UT : Utah<br>47 VT : Vermont<br>48 VA : Virginia<br>49 WA : Washington<br>50 WV : West Virginia<br>51 WI : Wisconsin<br>52 WY : Wyoming<br><br>Custom alignment: LV
6	res_program_state1<br><br>Show the field ONLY if: [residency_state]='1'	Please select your residency program.	dropdown (autocomplete)<br>1 University of Alabama Medical Center<br>2 University of South Alabama Hospitals<br><br>Custom alignment: LV
7	res_program_state2<br><br>Show the field ONLY if: [residency_state]='2'	Please select your residency program.	dropdown (autocomplete)<br>1 List of programs here<br><br>Custom alignment: LV
8	res_program_state3<br><br>Show the field ONLY if: [residency_state]='3'	Please select your residency program.	dropdown (autocomplete)<br>1 Creighton University School of Medicine/Maricopa Medical Center (Phoenix)<br>2 Midwestern University Osteopathic Postdoctoral Training Institute<br>3 University of Arizona

			College of Medicine at South Campus<br>4 University of Arizona College of Medicine-Tucson<br><br>Custom alignment: LV
9	res_program_state4<br><br>Show the field ONLY if: [residency_state]='4'	Please select your residency program.	dropdown (autocomplete)<br>1 Unity Health-White County Medical Center<br>2 University of Arkansas for Medical Sciences<br><br>Custom alignment: LV
10	res_program_state5<br><br>Show the field ONLY if: [residency_state]='5'	Please select your residency program.	dropdown (autocomplete)<br>1 Alameda Health System-Highland Hospital<br>2 Arrowhead Regional Medical Center<br>3 Desert Regional Medical Center<br>4 Eisenhower Medical Center<br>5 Kaiser Permanente Southern California<br>6 Kaweah Delta Health Care District (KDHCD)<br>7 Kern Medical Center<br>8 Loma Linda University Health Education Consortium<br>9 Los Angeles County-Harbor-UCLA Medical Center<br>10 Naval Medical Center (San Diego)<br>11 Riverside Community Hospital/University of California Riverside School of Medicine<br>12 St Joseph's Medical Center<br>13 Stanford Health Care-Sponsored Stanford University/Kaiser Permanente Medical Center

			14 UCLA David Geffen School of Medicine/UCLA Medical Center/Olive View<br>15 University of California (Irvine)<br>16 University of California (San Diego) Medical Center<br>17 University of California (San Francisco)/Fresno<br>18 University of California (San Francisco)/San Francisco General Hospital<br>19 University of California Davis Health<br>20 University of Southern California/LAC+USC Medical Center<br><br>Custom alignment: LV
11	res_program_state6<br><br>Show the field ONLY if:<br>[residency_state]='6'	Please select your residency program.	dropdown (autocomplete)<br>1 Denver Health Medical Center<br><br>Custom alignment: LV
12	res_program_state7<br><br>Show the field ONLY if:<br>[residency_state]='7'	Please select your residency program.	dropdown (autocomplete)<br>1 University of Connecticut<br>2 Yale-New Haven Medical Center<br><br>Custom alignment: LV
13	res_program_state8<br><br>Show the field ONLY if:<br>[residency_state]='8'	Please select your residency program.	dropdown (autocomplete)<br>1 Christiana Care Health Services<br><br>Custom alignment: LV
14	res_program_state9<br><br>Show the field ONLY if:<br>[residency_state]='9'	Please select your residency program.	dropdown (autocomplete)<br>1 George Washington University<br>2 MedStar Health/Georgetown-Washington Hospital

			Center<br><br>Custom alignment: LV
15	res_program_state10<br><br>Show the field ONLY if: [residency_state]='10'	Please select your residency program.	dropdown (autocomplete)<br>2 Aventura Hospital and Medical Center<br>3 Florida Atlantic University Charles E. Schmidt College of Medicine<br>4 Florida Hospital Medical Center<br>5 Florida State University College of Medicine<br>6 HCA West Florida GME Consortium/Brandon Regional Hospital<br>7 HCA West Florida GME Consortium/Oak Hill Hospital<br>8 Jackson Memorial Hospital/Jackson Health System<br>9 Kendall Regional Medical Center<br>10 Mount Sinai Medical Center of Florida, Inc.<br>11 Orange Park Medical Center<br>12 Orlando Health<br>13 Palm Beach Consortium for Graduate Medical Education<br>14 University of Central Florida/HCA GME Consortium (Gainesville)<br>15 University of Central Florida/HCA GME Consortium (Greater Orlando)<br>16 University of Central Florida/HCA GME Consortium (Ocala)<br>17 University of Florida<br>18 University of Florida

			College of Medicine Jacksonville<br>19 University of South Florida Morsani<br><br>Custom alignment: LV
16	res_program_state11<br><br>Show the field ONLY if: [residency_state]='11'	Please select your residency program.	dropdown (autocomplete)<br>1 Coliseum Medical Center<br>2 Emory University School of Medicine<br>3 Medical College of Georgia<br>4 WellStar Kennestone Regional Medical Center<br><br>Custom alignment: LV
17	res_program_state12<br><br>Show the field ONLY if: [residency_state]='12'	Please select your residency program.	dropdown (autocomplete)<br>1 List of programs here<br><br>Custom alignment: LV
18	res_program_state13<br><br>Show the field ONLY if: [residency_state]='13'	Please select your residency program.	dropdown (autocomplete)<br>1 List of programs here<br><br>Custom alignment: LV
19	res_program_state14<br><br>Show the field ONLY if: [residency_state]='14'	Please select your residency program.	dropdown (autocomplete)<br>1 Advocate Health Care (Advocate Christ Medical Center)<br>2 Cook County Health and Hospitals System<br>3 Loyola University Medical Center<br>4 McGaw Medical Center of Northwestern University<br>5 Presence Resurrection Medical Center<br>6 Rush University Medical Center<br>7 Southern Illinois University School of Medicine

			8 Swedish Covenant Hospital<br>9 University of Chicago<br>10 University of Illinois College of Medicine at Chicago<br>11 University of Illinois College of Medicine at Peoria<br><br>Custom alignment: LV
20	res_program_state15<br><br>Show the field ONLY if: [residency_state]='15'	Please select your residency program.	dropdown (autocomplete)<br>1 Indiana University School of Medicine<br><br>Custom alignment: LV
21	res_program_state16<br><br>Show the field ONLY if: [residency_state]='16'	Please select your residency program.	dropdown (autocomplete)<br>1 University of Iowa Hospitals and Clinics<br><br>Custom alignment: LV
22	res_program_state17<br><br>Show the field ONLY if: [residency_state]='17'	Please select your residency program.	dropdown (autocomplete)<br>1 University of Kansas School of Medicine<br><br>Custom alignment: LV
23	res_program_state18<br><br>Show the field ONLY if: [residency_state]='18'	Please select your residency program.	dropdown (autocomplete)<br>1 University of Kentucky College of Medicine<br>2 University of Louisville School of Medicine<br><br>Custom alignment: LV
24	res_program_state19<br><br>Show the field ONLY if: [residency_state]='19'	Please select your residency program.	dropdown (autocomplete)<br>1 Louisiana State University<br>2 Louisiana State University (Baton Rouge)<br>3 Louisiana State University (Shreveport)<br><br>Custom alignment: LV
25	res_program_state20	Please select your residency program.	dropdown (autocomplete)<br>1 Maine Medical Center

	Show the field ONLY if: [residency_state]='20'		Custom alignment: LV
26	res_program_state21<br><br>Show the field ONLY if: [residency_state]='21'	Please select your residency program.	dropdown (autocomplete)<br>1 Johns Hopkins University<br>2 University of Maryland<br><br>Custom alignment: LV
27	res_program_state22<br><br>Show the field ONLY if: [residency_state]='22'	Please select your residency program.	dropdown (autocomplete)<br>1 Beth Israel Deaconess Medical Center/Harvard Medical School<br>2 Boston University Medical Center<br>3 Massachusetts General Hospital/Brigham and Women's Hospital/Harvard Medical School<br>4 UMMS-Baystate<br>5 University of Massachusetts<br><br>Custom alignment: LV
28	res_program_state23<br><br>Show the field ONLY if: [residency_state]='23'	Please select your residency program.	dropdown (autocomplete)<br>1 Ascension Macomb-Oakland Hospital<br>2 Ascension St John Hospital<br>3 Beaumont Health (Farmington Hills)<br>4 Beaumont Health (Royal Oak)<br>5 Beaumont Health (Trenton and Dearborn)<br>6 Central Michigan University College of Medicine<br>7 Detroit Medical Center/Wayne State University (Detroit Receiving Hospital)<br>8 Detroit Medical Center/Wayne State University (Sinai Grace)

			9 Garden City Hospital<br>10 Genesys Regional Medical Center<br>11 Henry Ford Allegiance Health<br>12 Henry Ford Hospital/Wayne State University<br>13 Henry Ford Macomb Hospital<br>14 Henry Ford Wyandotte Hospital<br>15 Lakeland Health<br>16 McLaren Health Care/Macomb/MSU<br>17 McLaren Health Care/Oakland/MSU<br>18 Mercy Health<br>19 Metro Health University of Michigan Health (Metro Health)<br>20 ProMedica Monroe Regional Hospital<br>21 Sparrow Hospital/Michigan State University<br>22 Spectrum Health/Michigan State University<br>23 St Mary Mercy Hospital<br>24 University of Michigan Health System<br>25 Western Michigan University Homer Stryker MD School of Medicine<br><br>Custom alignment: LV
29	res_program_state24<br><br>Show the field ONLY if: [residency_state]='24'	Please select your residency program.	dropdown (autocomplete)<br>1 HealthPartners Institute/Regions Hospital<br>2 Hennepin Healthcare<br>3 Mayo Clinic College of Medicine and Science (Rochester)

			Custom alignment: LV
30	res_program_state25<br><br>Show the field ONLY if: [residency_state]='25'	Please select your residency program.	dropdown (autocomplete)<br>1 Merit Health Wesley<br>2 University of Mississippi Medical Center<br><br>Custom alignment: LV
31	res_program_state26<br><br>Show the field ONLY if: [residency_state]='26'	Please select your residency program.	dropdown (autocomplete)<br>1 Kansas City University of Medicine & Biosciences-GME Consortium (KCU-GME Consortium)/Freeman<br>2 St Louis University School of Medicine<br>3 University of Missouri-Columbia<br>4 University of Missouri-Kansas City School of Medicine<br>5 Washington University/B-JH/SLCH Consortium<br><br>Custom alignment: LV
32	res_program_state27<br><br>Show the field ONLY if: [residency_state]='27'	Please select your residency program.	dropdown (autocomplete)<br>1 List of programs here<br><br>Custom alignment: LV
33	res_program_state28<br><br>Show the field ONLY if: [residency_state]='28'	Please select your residency program.	dropdown (autocomplete)<br>1 University of Nebraska Medical Center<br><br>Custom alignment: LV
34	res_program_state29<br><br>Show the field ONLY if: [residency_state]='29'	Please select your residency program.	dropdown (autocomplete)<br>1 Sunrise Health GME Consortium<br>2 University of Nevada Las Vegas (UNLV) School of Medicine<br><br>Custom alignment: LV
35	res_program_state30<br><br>Show the field	Please select your residency program.	dropdown (autocomplete)<br>1 Mary Hitchcock Memorial

	ONLY if: [residency_state]='30'		Hospital/Dartmouth-Hitchcock<br><br>Custom alignment: LV
36	res_program_state31<br><br>Show the field ONLY if: [residency_state]='31'	Please select your residency program.	dropdown (autocomplete)<br>1 Atlantic Health (Morristown)<br>2 Cooper Medical School of Rowan University/Cooper University Hospital<br>3 Hackensack University Medical Center<br>4 Inspira Medical Center Woodbury<br>5 Newark Beth Israel Medical Center<br>6 Rowan SOM/Jefferson Health/Our Lady of Lourdes Health System<br>7 Rutgers New Jersey Medical School<br>8 Rutgers Robert Wood Johnson Medical School<br>9 St Joseph's University Medical Center<br><br>Custom alignment: LV
37	res_program_state32<br><br>Show the field ONLY if: [residency_state]='32'	Please select your residency program.	dropdown (autocomplete)<br>1 University of New Mexico School of Medicine<br><br>Custom alignment: LV
38	res_program_state33<br><br>Show the field ONLY if: [residency_state]='33'	Please select your residency program.	dropdown (autocomplete)<br>1 Albany Medical Center<br>2 Arnot Ogden Medical Center<br>3 Brookdale University Hospital and Medical Center<br>4 Brooklyn Hospital Center<br>5 Good Samaritan Hospital Medical Center<br>6 Icahn School of Medicine at Mount

	Sinai
7	Icahn School of Medicine at Mount Sinai (Beth Israel)
8	Icahn School of Medicine at Mount Sinai/St Luke's-Roosevelt Hospital Center
9	Lincoln Medical and Mental Health Center
10	Maimonides Medical Center
11	Montefiore Medical Center/Albert Einstein College of Medicine (Jacobi/Montefiore)
12	Nassau University Medical Center
13	New York Medical College (Metropolitan)
14	New York Presbyterian Hospital
15	New York University School of Medicine
16	New York-Presbyterian Brooklyn Methodist Hospital
17	New York-Presbyterian/Queens
18	NYCOMEC
19	St Barnabas Hospital
20	St. John's Riverside Hospital
21	Stony Brook Medicine/University Hospital
22	SUNY Health Science Center at Brooklyn
23	SUNY Upstate Medical University
24	University at Buffalo
25	University of Rochester
26	Wyckoff Heights Medical Center
27	Zucker School of Medicine at

			Hofstra/Northwell<br>28 Zucker School of Medicine at Hofstra/Northwell<br>29 Zucker School of Medicine at Hofstra/Northwell at Southside Hospital<br>30 Zucker School of Medicine at Hofstra/Northwell at Staten Island University Hospital<br><br>Custom alignment: LV
39	res_program_state34<br><br>Show the field ONLY if: [residency_state]='34'	Please select your residency program.	dropdown (autocomplete)<br>1 Campbell University/Cape Fear Valley Medical Center<br>2 Campbell University/Southeastern Regional Medical Center<br>3 Carolinas Medical Center<br>4 Duke University Hospital<br>5 University of North Carolina Hospitals<br>6 Vidant Medical Center/East Carolina University<br>7 Wake Forest University School of Medicine<br><br>Custom alignment: LV
40	res_program_state35<br><br>Show the field ONLY if: [residency_state]='35'	Please select your residency program.	dropdown (autocomplete)<br>1 List of programs here<br><br>Custom alignment: LV
41	res_program_state36<br><br>Show the field ONLY if: [residency_state]='36'	Please select your residency program.	dropdown (autocomplete)<br>1 Adena Regional Medical Center<br>2 Akron General Medical Center/NEOMED<br>3 Case Western Reserve University (MetroHealth)

			4 Case Western Reserve University/University Hospitals Cleveland Medical Center<br>5 Doctors Hospital/OhioHealth<br>6 Kettering Health Network<br>7 Memorial Health System<br>8 Mercy St Vincent Medical Center/Mercy Health Partners<br>9 Ohio State University Hospital<br>10 St Elizabeth Boardman Hospital<br>11 University Hospitals Osteopathic Consortium (UHOC)<br>12 University of Cincinnati Medical Center/College of Medicine<br>13 University of Toledo<br>14 Wright State University<br><br>Custom alignment: LV
42	res_program_state37<br><br>Show the field ONLY if: [residency_state]='37'	Please select your residency program.	dropdown (autocomplete)<br>1 Integris Health<br>2 Oklahoma State University Center for Health Sciences (Lawton)<br>3 Oklahoma State University Center for Health Sciences (Norman)<br>4 Oklahoma State University Center for Health Sciences (Tulsa)<br>5 University of Oklahoma School of Community Medicine (Tulsa)<br><br>Custom alignment: LV
43	res_program_state38	Please select your residency program.	dropdown (autocomplete)<br>1 Oregon Health &

	Show the field ONLY if: [residency_state]='38'		Science University<br><br>Custom alignment: LV
44	res_program_state39<br><br>Show the field ONLY if: [residency_state]='39'	Please select your residency program.	dropdown (autocomplete)<br>1 Albert Einstein Medical Center<br>2 Allegheny Health Network Medical Education Consortium<br>3 Allegheny Health Network Medical Education Consortium (AGH)<br>4 Conemaugh Memorial Medical Center<br>5 Crozer-Chester Medical Center<br>6 Drexel University College of Medicine/Hahnemann University Hospital<br>7 Geisinger Health System<br>8 Jefferson Health Northeast<br>9 Lehigh Valley Health Network/University of South Florida College of Medicine<br>10 Penn State Milton S Hershey Medical Center<br>11 Reading Hospital<br>12 Robert Packer Hospital<br>13 Sidney Kimmel Medical College at Thomas Jefferson University/TJUH<br>14 St Luke's Hospital<br>15 Temple University Hospital<br>16 University of Pennsylvania Health System<br>17 UPMC Medical Education (Erie)<br>18 UPMC Medical

			Education (Pittsburgh)<br>19 UPMC Pinnacle Hospitals<br>20 York Hospital<br><br>Custom alignment: LV
45	res_program_state40<br><br>Show the field ONLY if: [residency_state]='40'	Please select your residency program.	dropdown (autocomplete)<br>1 Hospital Episcopal San Lucas/Ponce School of Medicine<br>2 University of Puerto Rico<br><br>Custom alignment: LV
46	res_program_state41<br><br>Show the field ONLY if: [residency_state]='41'	Please select your residency program.	dropdown (autocomplete)<br>1 Brown University<br>2 Kent Hospital<br><br>Custom alignment: LV
47	res_program_state42<br><br>Show the field ONLY if: [residency_state]='42'	Please select your residency program.	dropdown (autocomplete)<br>1 Grand Strand Regional Medical Center<br>2 Greenville Health System/University of South Carolina<br>3 Medical University of South Carolina<br>4 Palmetto Health/University of South Carolina School of Medicine<br><br>Custom alignment: LV
48	res_program_state43<br><br>Show the field ONLY if: [residency_state]='43'	Please select your residency program.	dropdown (autocomplete)<br>1 List of programs here<br><br>Custom alignment: LV
49	res_program_state44<br><br>Show the field ONLY if: [residency_state]='44'	Please select your residency program.	dropdown (autocomplete)<br>1 University of Tennessee College of Medicine at Chattanooga<br>2 University of Tennessee College of Medicine at Memphis<br>3 University of Tennessee

			College of Medicine at Murfreesboro<br>4 Vanderbilt University Medical Center<br><br>Custom alignment: LV
50	res_program_state45<br><br>Show the field ONLY if: [residency_state]='45'	Please select your residency program.	dropdown (autocomplete)<br>1 Baylor College of Medicine<br>2 Christus Health/Texas A&M College of Medicine/Spohn Hospital<br>3 Darnall Army Medical Center<br>4 John Peter Smith Hospital (Tarrant County Hospital District)<br>5 San Antonio Uniformed Services Health Education Consortium<br>6 Texas A&M College of Medicine-Scott and White Medical Center (Temple)<br>7 Texas Tech University Health Sciences Center at Lubbock<br>8 Texas Tech University Health Sciences Center-PLFSOM<br>9 University of Texas at Austin Dell Medical School<br>10 University of Texas Health Science Center at Houston<br>11 University of Texas Health Science Center San Antonio Joe and Teresa Lozano Long School of Medicine<br>12 University of Texas Southwestern Medical School<br><br>Custom alignment: LV
51	res_program_state46	Please select your residency program.	dropdown (autocomplete)<br>1 University of Utah

	Show the field ONLY if: [residency_state]='46'		Custom alignment: LV
52	res_program_state47<br><br>Show the field ONLY if: [residency_state]='47'	Please select your residency program.	dropdown (autocomplete)<br>1 University of Vermont Medical Center<br><br>Custom alignment: LV
53	res_program_state48<br><br>Show the field ONLY if: [residency_state]='48'	Please select your residency program.	dropdown (autocomplete)<br>1 Carilion Clinic-Virginia Tech Carilion School of Medicine<br>2 Eastern Virginia Medical School<br>3 Naval Medical Center (Portsmouth)<br>4 University of Virginia Medical Center<br>5 Virginia Commonwealth University Health System<br><br>Custom alignment: LV
54	res_program_state49<br><br>Show the field ONLY if: [residency_state]='49'	Please select your residency program.	dropdown (autocomplete)<br>1 Madigan Healthcare System<br>2 University of Washington<br><br>Custom alignment: LV
55	res_program_state50<br><br>Show the field ONLY if: [residency_state]='50'	Please select your residency program.	dropdown (autocomplete)<br>2 Charleston Area Medical Center<br>3 Mountain State Osteopathic Postdoctoral Training Institutions, Inc. (MSOPTI)/Ohio Valley Medical Center<br>4 West Virginia University<br><br>Custom alignment: LV
56	res_program_state51<br><br>Show the field ONLY if: [residency_stat	Please select your residency program.	dropdown (autocomplete)<br>1 Medical College of Wisconsin Affiliated Hospitals<br>2 University of Wisconsin

	e]='51'		| | Hospitals and Clinics | <br>Custom alignment: LV
57	res_program_state52<br><br>Show the field ONLY if: [residency_state]='52'	Please select your residency program.	dropdown (autocomplete)<br>1 List of programs here<br><br>Custom alignment: LV
58	combined_spec<br><br>Show the field ONLY if: [em_residncy] = '1'	Was that training part of a combined specialty training program?	yesno<br>1 Yes<br>0 No<br><br>Custom alignment: LV
59	other_spec<br><br>Show the field ONLY if: [combined_spec] = '1'	What is the other specialty combined with emergency medicine in your program?	text<br>Custom alignment: LV
60	fellship_program<br><br>Show the field ONLY if: [em_residncy] = '2'	Which fellowship program are you attending?	text<br>Custom alignment: LV
61	fellowship_type<br><br>Show the field ONLY if: [em_residncy] = '2'	What type of fellowship program are you completing?	radio<br>1 Emergency Medical Services<br>2 Medical Toxicology<br>3 Pediatric Emergency Medicine<br>4 Sports Medicine<br>5 Undersea and Hyperbaric Medicine<br>6 Clinical Informatics<br><br>Custom alignment: LV
62	gender	Section Header: *DEMOGRAPHICS*<br><br>What is your gender?	radio<br>1 Female<br>2 Male<br>3 Prefer to self-describe<br>4 Prefer not to answer<br><br>Custom alignment: LV
63	age	How old are you?	dropdown

65	< 25
25	25
26	26
27	27
28	28
29	29
30	30
31	31
32	32
33	33
34	34
35	35
36	36
37	37
38	38
39	39
40	40
41	41
42	42
43	43
44	44
45	45
46	46
47	47
48	48
49	49
50	50
51	51
52	52
53	53
54	54
55	55
56	56
57	57
58	58
59	59
60	60

			61 61<br>62 62<br>63 63<br>64 64<br>66 65+<br><br>Custom alignment: LV
64	citizenship	What is your current citizenship status?	radio<br>1 Native born U.S. citizen<br>2 Naturalized U.S. citizen<br>3 Permanent resident<br>4 H-1, H-2, or H-3 visa (temporary worker)<br>5 J-1 or J-2 visa (exchange visitor)<br>6 Other visa<br><br>Custom alignment: LV
65	citizenship_other<br><br>Show the field ONLY if:<br>[citizenship]="6"	Please specify your "other" current visa status	text<br>Custom alignment: LV
66	hispanic_latino	Are you Hispanic/Latino/Latina?	radio<br>1 Yes<br>2 No<br><br>Custom alignment: LV
67	race	What is your race? (Select all that apply.)	checkbox<br>1 race___1 American Indian/Alaska Native<br>2 race___2 Asian or Pacific Islander<br>3 race___3 Black/African American<br>4 race___4 White<br>5 race___5 Other<br><br>Custom alignment: LV
68	race_other<br><br>Show the field ONLY if:	Please specify.	text<br>Custom alignment: LV

	[race(5)] = '1'		
69	secondary_ed_location	Where did you live when you completed your secondary education (i.e., high school)?	radio<br>1 U.S.<br>2 Canada<br>3 Other country<br><br>Custom alignment: LV
70	med_education_type	What type of medical education do you have?	radio<br>1 Allopathic (MD, MBBS)<br>2 Osteopathic (DO)<br><br>Custom alignment: LV
71	med_school_location	Where did you attend medical school?	radio<br>1 U.S.<br>2 Canada<br>3 Other country<br><br>Custom alignment: LV
72	em_training_years	At the end of the 2018-2019 training year, how many total years of Emergency Medicine training will you have completed?	radio, Required<br>1 1<br>2 2<br>3 3<br>4 4<br>5 5 or more<br><br>Custom alignment: LV
73	prior_gme	Did you have any graduate medical education (GME) prior to your training in emergency medicine?	yesno<br>1 Yes<br>0 No<br><br>Custom alignment: LV
74	prior_gme_speclty<br><br>Show the field ONLY if:<br>[prior_gme]='1'	In what specialty was your prior GME training?	text<br>Custom alignment: LV
75	when_oth_spec_start<br><br>Show the field ONLY if:<br>[prior_gme]='1'	In which year did you begin your prior GME training?	text (number, Min: 1960, Max: 2020)
76	other_spec_complete	Did you complete your prior GME training?	yesno<br>1 Yes

	Show the field ONLY if: [prior_gme]='1'		0 No
77	when_oth_spec_end<br>Show the field ONLY if: [prior_gme]='1'	In which year did your prior GME training end?	text (integer, Min: 1960, Max: 2020)
78	prior_degrees	Do you have any other post-college degrees (check all that apply)?	checkbox<br>1 prior_degrees___1 No<br>2 prior_degrees___2 Yes, MBA<br>3 prior_degrees___3 Yes, MPH<br>4 prior_degrees___4 Yes, PhD<br>5 prior_degrees___5 Yes, other degree (please describe below)<br>Custom alignment: LV
79	prior_degrees_other<br>Show the field ONLY if: [prior_degrees(5)] = '1'	Please describe your "other" prior post-college degree(s).	text<br>Custom alignment: LV
80	ed_debt_level	What is your current level of educational debt from college and medical school?	dropdown<br>1 None<br>2 Less than $25,000<br>3 $25,000-$49,999<br>4 $50,000-$74,999<br>5 $75,000-$99,999<br>6 $100,000-$124,999<br>7 $125,000-$149,999<br>8 $150,000-$174,999<br>9 $175,000-$199,999<br>10 $200,000-$224,999<br>11 $225,000-$249,999<br>12 $250,000-$274,999<br>13 $275,000-$299,999

			14 | $300,000 and over<br><br>Custom alignment: LV
81	end_year_activity	Section Header: *FUTURE PLANS*<br>What will your primary activity be at the end of the 2018-2019 training year? (Select one.)	radio<br>1 | Clinical practice<br>2 | Continue current residency<br>3 | Emergency Medicine subspecialty or other additional training<br>4 | Teaching/research (in non-training position)<br>6 | Temporarily out of medicine<br>7 | Undecided/don't know yet<br>8 | Other<br><br>Custom alignment: LV
82	end_year_activity_other<br><br>Show the field ONLY if: [end_year_activity] = '8'	Please specify.	text<br>Custom alignment: LV
83	addl_training<br><br>Show the field ONLY if: [end_year_activity] = '3'	What additional training are you pursuing? (Select one.)	radio<br>1 | Emergency Medical Services<br>2 | Medical Toxicology<br>3 | Pediatric Emergency Medicine<br>4 | Sports Medicine<br>5 | Undersea and Hyperbaric Medicine<br>6 | Clinical Informatics<br>7 | Advanced Emergency Medicine<br>8 | Critical Care Medicine<br>9 | Hospice and Palliative Medicine<br>10 | Pain Management<br>11 | Ultrasonography<br>12 | Other (please specify below)

			Custom alignment: LV
84	addl_training_other<br>Show the field ONLY if: [addl_training] = '12'	Please specify.	text<br>Custom alignment: LV
85	job_search<br>Show the field ONLY if: [em_training_years] = '2' or [em_training_years] = '3' or [em_training_years] = '4' or [em_training_years]='5'	Section Header: *JOB MARKET EXPERIENCES*<br>Have you searched for an Emergency Medicine job beginning after the 2018-2019 training year?	radio<br>1 Yes<br>2 No, not yet<br>3 No, and I do not plan to do so<br>Custom alignment: LV
86	why_not_searched<br>Show the field ONLY if: [job_search]='2' or [job_search]='3'	Why have you not searched for an emergency medicine job?	text<br>Custom alignment: LV
87	considered_rural<br>Show the field ONLY if: [job_search] = '1'	Did you consider looking for a job or position in a rural or semi-rural community?	yesno<br>1 Yes<br>0 No<br>Custom alignment: LV
88	why_not_rural<br>Show the field ONLY if: [considered_rural]='0'	Why did you not consider looking for a job in a rural or semi-rural area (check all that apply)?	checkbox<br>1 why_not_rural___1 Life style/social life concerns<br>2 why_not_rural___2 Concern with low patient volume and/or nature of EM practice<br>3 why_not_rural___3 Lack of peers<br>4 why_not_rural___4 Limited career advancement opportunities<br>5 why_not_rural___5 Limited spousal job opportunities<br>6 why_not_rural___6 Limited school

			(continued) options for children<br>7 why_not_rural___7 Other<br>Custom alignment: LV
89	why_not_rural_other<br>Show the field ONLY if: [why_not_rural(7)]='1'	Please describe your "other" reason(s) for not considering a rural or semi-rural position.	text<br>Custom alignment: LV
90	job_offer	Have you been offered an Emergency Medicine job beginning after the 2018-2019 training year?	radio<br>1 Yes, and I have accepted an offer<br>2 Yes, but I am still searching<br>3 No, I have not yet been offered a job<br>Custom alignment: LV
91	job_search_difficulty<br>Show the field ONLY if: [job_search] = '1'	Did you have difficulty finding an Emergency Medicine job you were satisfied with?	radio<br>1 Yes<br>2 No<br>Custom alignment: LV
92	job_search_difficulty_rsn<br>Show the field ONLY if: [job_search_difficulty] = '1'	What would you say was the main reason you had difficulty? (Select one.)	radio<br>1 Overall lack of jobs/practice opportunities<br>2 Lack of jobs/practice opportunities in desired locations<br>3 Lack of jobs/practice opportunities in desired practice setting (e.g., trauma level I hospital)<br>4 Inadequate salary/compensation<br>5 Lack of jobs/practice opportunities that meet visa status requirements<br>6 Lack of employment opportunities for spouse/partner<br>7 Other<br>Custom alignment: LV

93	job_search_difficulty_reason_other<br><br>Show the field ONLY if: [job_search_difficulty_rsn] = '7'	Please specify.	text<br>Custom alignment: LV
94	change_plans<br><br>Show the field ONLY if: [job_search] = '1'	Did you have to change your plans because of limited Emergency Medicine job opportunities?	radio<br>1 Yes<br>2 No<br><br>Custom alignment: LV
95	job_applications<br><br>Show the field ONLY if: [job_search] = '1'	How many jobs did you apply for (excluding residencies and other training positions)?	radio<br>6 None<br>1 1<br>2 2<br>3 3<br>4 4<br>5 5<br>7 6-10<br>8 Over 10<br><br>Custom alignment: LV
96	job_offers<br><br>Show the field ONLY if: [job_search] = '1'	How many Emergency Medicine job offers did you receive (excluding residencies and other training positions)?	radio<br>6 None<br>1 1<br>2 2<br>3 3<br>4 4<br>5 5<br>7 6-10<br>8 Over 10<br><br>Custom alignment: LV
97	more_available_jobs<br><br>Show the field ONLY if: [job_search] = 1	Were there certain types of EM positions or settings that were more available than others, e.g. urban/rural, teaching/non-teaching hospital, trauma level of hospitals, positions with large national firms?	radio<br>1 Yes<br>2 No<br><br>Custom alignment: LV
98	more_available_jobs_des<br><br>Show the field ONLY if:	Please describe the types of EM positions or settings that were more available than others.	notes<br>Custom alignment: LV

	[more_available_jobs]=1		
99	less_available_jobs<br>Show the field ONLY if: [job_search]=1	Were there certain types of Emergency Medicine positions or settings that were less available than others?	radio<br>1 Yes<br>2 No<br>Custom alignment: LV
100	less_available_jobs_des<br>Show the field ONLY if: [less_available_jobs]=1	Please describe the types of EM positions or settings that were less available than others.	notes<br>Custom alignment: LV
101	primary_clinical_focus<br>Show the field ONLY if: [job_offer] = '1'	Section Header: *JOB CHARACTERISTICS*<br>Which of the following best describes the focus of your primary post-training clinical position? (Select one.)	radio<br>1 Emergency medicine<br>2 Mixed emergency medicine and critical care<br>3 Other clinical specialty area<br>4 Other, please describe below<br>Custom alignment: LV
102	primary_clinical_focus_other<br>Show the field ONLY if: [primary_clinical_focus] = '4'	Please specify.	text<br>Custom alignment: LV
103	primary_job_orgzn<br>Show the field ONLY if: [job_offer] = '1'	Which of the following best describes your organizational arrangement in your primary post-training Emergency Medicine position?	radio<br>1 Employee of hospital<br>2 Employee of group practice at hospital<br>3 Employee of a local organization that contracts with hospital(s) I work at<br>4 Employee of a multi-state organization that employees Emergency Medicine Physicians<br>5 Employee of university<br>6 Independent contractor<br>7 Locum Tenens<br>8 Other (please describe below)

			Custom alignment: LV
104	primary_job_orgzn_other<br>Show the field ONLY if: [primary_job_orgzn] = '8'	Please specify.	text<br>Custom alignment: LV
105	primary_job_setting<br>Show the field ONLY if: [job_offer] = '1'	Which of the following best describes the setting where you will be spending most of your time?	radio<br>1 Trauma level I hospital<br>2 Trauma level II hospital<br>3 Trauma level III hospital<br>4 Trauma level IV hospital<br>5 Trauma level V hospital<br>6 Other hospital<br>7 Urgent care facility<br>8 Free-standing ED<br>9 Other (please specify)<br>Custom alignment: LV
106	other_hospital_type<br>Show the field ONLY if: [primary_job_setting] = '6'	Is this hospital a:	radio<br>1 Critical Access Hospital<br>2 Rural Referral Center<br>3 Sole Community Hospital<br>4 Medicare Dependent Hospital<br>5 Disproportionate Share Hospital<br>6 Other, or do not know<br>Custom alignment: LV
107	primary_job_setting_other<br>Show the field ONLY if: [primary_job_setting] = '9'	Please specify.	text<br>Custom alignment: LV
108	acad_med_ctr<br>Show the field ONLY if: [job_offer] = '1'	Will your position be at an academic medical center?	yesno<br>1 Yes<br>0 No<br>Custom alignment: LV
109	supervis_resdnt	Will you be involved in supervising	yesno

	s<br><br>Show the field ONLY if:<br>[job_offer] = '1'	EM residents?	1 Yes<br>0 No<br><br>Custom alignment: LV
110	whether_for_profit<br><br>Show the field ONLY if:<br>[job_offer]='1'	Will your post-training employer be:	radio<br>1 A not-for-profit organization<br>2 A for-profit organization<br>3 I don't know<br><br>Custom alignment: LV
111	number_hospitals<br><br>Show the field ONLY if:<br>[job_offer]='1'	In your primary post-training EM position, how many different hospitals do you expect to work in during a typical month?	text (integer, Min: 0, Max: 99)<br>Custom alignment: LV
112	paid_weeklyhrs<br><br>Show the field ONLY if:<br>[job_offer] = '1'	What is your typical number of paid hours per week?	text (integer, Min: 0, Max: 100)<br>Custom alignment: LV
113	fte<br><br>Show the field ONLY if:<br>[job_offer] = '1'	Is your position a full time one?	yesno<br>1 Yes<br>0 No<br><br>Custom alignment: LV
114	fte_pref<br><br>Show the field ONLY if:<br>[fte]='0'	Was it your preference not to work full time?	yesno<br>1 Yes<br>0 No<br><br>Custom alignment: LV
115	fte_pcnt<br><br>Show the field ONLY if:<br>[fte] = '0'	What percentage of a full-time equivalent position (FTE, however that is defined in your organization) is your employment contract for?	radio<br>1 90-99%<br>2 80-89%<br>3 70-79%<br>4 60-69%<br>5 50-59%<br>6 40-49%<br>7 30-39%<br>8 20-29%<br>9 10-19%<br>10 0-9%<br>11 It's more complicated than that (please

			describe)<br><br>Custom alignment: LV
116	fte_expl<br><br>Show the field ONLY if: [fte_pcnt] = '11'	Please describe the nature of your position(s) if percentage of FTE is not an adequate way to describe it.	notes<br>Custom alignment: LV
117	patient_types<br><br>Show the field ONLY if: [job_offer]='1'	In your post-training position(s), which of the following patient types do you expect to be treating (check all that apply)?	checkbox<br>1 patient_types___1 Pediatric<br>2 patient_types___2 Geriatric<br>3 patient_types___3 Trauma<br>4 patient_types___4 Stroke<br>5 patient_types___5 Behavioral health<br>6 patient_types___6 Substance abuse<br>7 patient_types___7 Prisoners<br>8 patient_types___8 Other special populations (please describe below)<br><br>Custom alignment: LV
118	patient_types_other<br><br>Show the field ONLY if: [patient_types(8)]='1'	Please specify the other special patient population(s) you expect to be working with.	text<br>Custom alignment: LV
119	secondary_job<br><br>Show the field ONLY if: [job_offer] = '1'	Outside of your primary Emergency Medicine job responsibilities, do you expect to hold a secondary position (e.g., moonlighting, hospitalist)?	yesno<br>1 Yes<br>0 No<br><br>Custom alignment: LV
120	secondary_job_describe<br><br>Show the field ONLY if: [secondary_job] ='1'	Please describe the secondary position(s) you expect to hold in addition to your primary Emergency Medicine position.	text<br>Custom alignment: LV
121	whether_primary_job_us<br><br>Show the field ONLY if: [job_offer]='1'	Section Header: *JOB LOCATION AND SERVICE REQUIREMENT*<br><br>Is your primary Emergency Medicine job located in the United States?	yesno<br>1 Yes<br>0 No

			Custom alignment: LV
122	primary_job_state<br><br>Show the field ONLY if: [whether_primary_job_us]='1'	Which state is your primary Emergency Medicine position located in?	dropdown (autocomplete)

1	AL : Alabama
2	AK : Alaska
3	AZ : Arizona
4	AR : Arkansas
5	CA : California
6	CO : Colorado
7	CT : Connecticut
8	DE : Delaware
9	DC : District of Colombia
10	FL : Florida
11	GA : Georgia
12	HI : Hawaii
13	ID : Idaho
14	IL : Illinois
15	IN : Indiana
16	IA : Iowa
17	KS : Kansas
18	KY : Kentucky
19	LA : Louisiana
20	ME : Maine
21	MD : Maryland
22	MA : Massachusetts
23	MI : Michigan
24	MN : Minnesota
25	MS : Mississippi
26	MO : Missouri
27	MT : Montana
28	NE : Nebraska
29	NV : Nevada
30	NH : New Hampshire
31	NJ : New Jersey
32	NM : New Mexico
33	NY : New York
34	NC : North Carolina
35	ND : North Dakota

			36 OH : Ohio<br>37 OK : Oklahoma<br>38 OR : Oregon<br>39 PA : Pennsylvania<br>40 PR : Puerto Rico<br>41 RI : Rhode Island<br>42 SC : South Carolina<br>43 SD : South Dakota<br>44 TN : Tennessee<br>45 TX : Texas<br>46 UT : Utah<br>47 VT : Vermont<br>48 VA : Virginia<br>49 WA : Washington<br>50 WV : West Virginia<br>51 WI : Wisconsin<br>52 WY : Wyoming<br><br>Custom alignment: LV
123	primary_job_zip_code<br><br>Show the field ONLY if: [whether_primary_job_us]='1'	What is the 5 digit zip code of the practice address of your primary Emergency Medicine job?<br><br>If unknown please enter city and state.	text, Identifier<br>Custom alignment: LV
124	whether_obligation<br><br>Show the field ONLY if: [job_offer]='1'	Do you have a federal, regional or state service obligation to work for a minimum number of years in particular communities or settings?	yesno<br>1 Yes<br>0 No<br><br>Custom alignment: LV
125	service_obligation<br><br>Show the field ONLY if: [whether_obligation]='1'	Which of the following best describes the reason for your service obligation?	radio<br>1 Visa waiver program<br>2 National Health Service Corps or other loan forgiveness program<br>3 Military<br>4 Other<br><br>Custom alignment: LV
126	loan_forgive_prog	Which loan forgiveness program are you enrolling in?	radio<br>1 National Health Service Corps

	Show the field ONLY if: [service_obligation]='2'		2 State loan forgiveness program<br>3 Other loan forgiveness program<br><br>Custom alignment: LV
127	obligation_years<br><br>Show the field ONLY if: [whether_obligation]='1'	What is the length of your service period obligation?	radio<br>1 1 year<br>2 2 years<br>3 3 years<br>4 4 years<br>5 5 years<br>6 More than 5 years<br><br>Custom alignment: LV
128	buy_out<br><br>Show the field ONLY if: [whether_obligation]='1'	Do you plan to buy out your service obligation?	radio<br>1 Yes,<br>2 No<br>3 Not sure yet<br><br>Custom alignment: LV
129	primary_job_years<br><br>Show the field ONLY if: [job_offer] = '1'	How many years do you expect to be at your primary Emergency Medicine position?	radio<br>1 1<br>2 2<br>3 3<br>4 4<br>5 5<br>6 6 or more<br><br>Custom alignment: LV
130	primary_job_demog<br><br>Show the field ONLY if: [job_offer] = '1'	Which best describes the demographics of the area of your primary Emergency Medicine job?	radio<br>1 Large city (population over 1 million)<br>2 Medium city (population over 250,000 but less than 1 million)<br>3 Small city (population over 50,000 but less than 250,000)<br>4 Semi-rural (population over 2,500 but less than 50,000)

			5 Rural<br><br>Custom alignment: LV
131	primary_job_em_visits<br><br>Show the field ONLY if: [job_offer] = '1'	If your primary Emergency Medicine position is in a hospital, how many emergency visits were made in 2018 at this hospital (leave blank if you will not be working in a hospital)?	text (integer, Min: 0)<br>Custom alignment: LV
132	primary_job_em_physicians<br><br>Show the field ONLY if: [job_offer] = '1'	If your primary Emergency Medicine position is in a hospital, what is the estimated number of full time equivalent emergency physicians working in the Emergency Department (leave blank if you will not be working in a hospital)?	text (integer, Min: 0)<br>Custom alignment: LV
133	work_with_nps<br><br>Show the field ONLY if: [job_offer] = '1'	Will you be supervising an NP in your new job?	radio<br>1 Yes<br>2 No<br>3 Don't know<br><br>Custom alignment: LV
134	work_with_pas<br><br>Show the field ONLY if: [job_offer] = '1'	Will you be supervising a PA in your new job?	radio<br>1 Yes<br>2 No<br>3 Don't know<br><br>Custom alignment: LV
135	prim_job_hrs_ptcare<br><br>Show the field ONLY if: [job_offer] = '1'	Section Header: *In your primary emergency medicine position, how many hours per week do you expect to spend in each of the following activities?*<br><br>Direct patient care	radio (Matrix)<br>1 None<br>2 1 to 9<br>3 10 to 19<br>4 20 to 29<br>5 30 to 39<br>6 40 to 49<br>7 50 to 59<br>8 60+
136	prim_job_hrs_research<br><br>Show the field ONLY if: [job_offer] = '1'	Research	radio (Matrix)<br>1 None<br>2 1 to 9<br>3 10 to 19<br>4 20 to 29<br>5 30 to 39

			6 40 to 49<br>7 50 to 59<br>8 60+
137	prim_job_hrs_teaching<br><br>Show the field ONLY if: [job_offer] = '1'	Teaching	radio (Matrix)<br>1 None<br>2 1 to 9<br>3 10 to 19<br>4 20 to 29<br>5 30 to 39<br>6 40 to 49<br>7 50 to 59<br>8 60+
138	prim_job_hrs_admin<br><br>Show the field ONLY if: [job_offer] = '1'	Administration	radio (Matrix)<br>1 None<br>2 1 to 9<br>3 10 to 19<br>4 20 to 29<br>5 30 to 39<br>6 40 to 49<br>7 50 to 59<br>8 60+
139	prim_job_hrs_service<br><br>Show the field ONLY if: [job_offer] = '1'	Volunteering/community service	radio (Matrix)<br>1 None<br>2 1 to 9<br>3 10 to 19<br>4 20 to 29<br>5 30 to 39<br>6 40 to 49<br>7 50 to 59<br>8 60+
140	prim_job_incentives<br><br>Show the field ONLY if: [job_offer] = '1'	Please identify all of the incentives you received for accepting your primary Emergency Medicine job. (Select all that apply.)	checkbox<br>1 prim_job_incentives___1 Sign-on bonus<br>2 prim_job_incentives___2 Income guarantees<br>3 prim_job_incentives___3 Night shift bonuses<br>4 prim_job_incentives___4 Night shift differential

			5 | prim_job_incentives___5 | Relocation allowances<br>6 | prim_job_incentives___6 | Spouse/partner job transition assistance<br>7 | prim_job_incentives___7 | Support for maintenance of certification and continuing medical education<br>8 | prim_job_incentives___8 | Career development opportunities<br>9 | prim_job_incentives___9 | Educational loan repayment<br>10 | prim_job_incentives___10 | H-1 visa sponsorship<br>11 | prim_job_incentives___11 | J-1 visa waiver<br>12 | prim_job_incentives___12 | Other<br><br>Custom alignment: LV
141	prim_job_incentives_other<br><br>Show the field ONLY if: [prim_job_incentives(12)] = '1'	Please specify.	text<br>Custom alignment: LV
142	partner_poss<br><br>Show the field ONLY if: [job_offer]='1'	In your post training position can you become a partner in the future?	radio<br>1 | Yes<br>2 | No<br>3 | Already a partner<br><br>Custom alignment: LV
143	partner_poss_years<br><br>Show the field ONLY if: [partner_poss]='1'	What is the minimum years of practice required before being eligible for partner?	text (integer, Min: 0, Max: 99)<br>Custom alignment: LV
144	prim_job_base_salary<br><br>Show the field ONLY if: [job_offer] = '1'	What is your expected base salary from your primary Emergency Medicine job during your first year of practice?	dropdown<br>1 | Less than $50,000<br>2 | $50,000 - $59,999<br>3 | $60,000 - $69,999<br>4 | $70,000 - $79,999

5	$80,000 - $89,999
6	$90,000 - $99,999
7	$100,000 - $109,999
8	$110,000 - $119,999
9	$120,000 - $129,999
10	$130,000 - $139,999
11	$140,000 - $149,999
12	$150,000 - $159,999
13	$160,000 - $169,999
14	$170,000 - $179,999
15	$180,000 - $189,999
16	$190,000 - $199,999
17	$200,000 - $209,999
18	$210,000 - $219,999
19	$220,000 - $229,999
20	$230,000 - $239,999
21	$240,000 - $249,999
22	$250,000 - $259,999
23	$260,000 - $269,999
24	$270,000 - $279,999
25	$280,000 - $289,999
26	$290,000 - $299,999
27	$300,000 - $309,999
28	$310,000 - $319,999
29	$320,000 - $329,999
30	$330,000 - $339,999
31	$340,000 - $349,999
32	$350,000 - $359,999
33	$360,000 - $369,999
34	$370,000 - $379,999
35	$380,000 - $389,999
36	$390,000 - $399,999
37	$400,000 - $409,999
38	$410,000 - $419,999
39	$420,000 - $429,999
40	$430,000 - $439,999
41	$440,000 - $449,999

			42 $450,000 - $459,999<br>43 $460,000 - $469,999<br>44 $470,000 - $479,999<br>45 $480,000 - $489,999<br>46 $490,000 - $499,999<br>47 $500,000 or over<br><br>Custom alignment: LV
145	prim_job_perfmce_income<br><br>Show the field ONLY if: [job_offer]='1'	Will you receive additional income based on performance?	yesno<br>1 Yes<br>0 No<br><br>Custom alignment: LV
146	comp_mods<br><br>Show the field ONLY if: [prim_job_perfmce_income]='1'	What modifiers does your compensation include (select all that apply)?	checkbox<br>1 comp_mods___1 Number of patients seen<br>2 comp_mods___2 Patients seen per hour<br>3 comp_mods___3 RVUs<br>4 comp_mods___4 Patient satisfaction<br>5 comp_mods___5 Other<br><br>Custom alignment: LV
147	comp_mods_other<br><br>Show the field ONLY if: [comp_mods(5)]='1'	Please describe the other modifier(s) applying to your compensation.	text<br>Custom alignment: LV
148	prim_job_addl_income<br><br>Show the field ONLY if: [prim_job_perfmce_income]='1'	What is your expected additional incentive income from your primary Emergency Medicine job during your first year of practice?	dropdown<br>2 Less than $5,000<br>3 $5,000-$9,999<br>4 $10,000-$14,999<br>5 $15,000-$19,999<br>6 $20,000-$24,999<br>7 $25,000-$29,999<br>8 $30,000-$34,999<br>9 $35,000-$39,999<br>10 $40,000-$44,999<br>11 $45,000-$49,999

			12 $50,000-$54,999<br>13 $55,000-$59,999<br>14 $60,000 or more<br>15 Unknown<br><br>Custom alignment: LV
149	salary_comp_satisfaction<br><br>Show the field ONLY if: [job_offer] = '1'	What is your level of satisfaction with your overall salary/compensation?	radio<br>1 Very satisfied<br>2 Somewhat satisfied<br>3 Somewhat dissatisfied<br>4 Very dissatisfied<br><br>Custom alignment: LV
150	job_satisfaction<br><br>Show the field ONLY if: [job_offer] = '1'	What is your overall level of satisfaction with your new job?	checkbox<br>1 job_satisfaction___1 Very satisfied<br>2 job_satisfaction___2 Somewhat satisfied<br>3 job_satisfaction___3 Somewhat dissatisfied<br>4 job_satisfaction___4 Very dissatisfied<br><br>Custom alignment: LV
151	imp_start_time<br><br>Show the field ONLY if: [job_offer] = '1'	Section Header: *Please rate the importance of the following factors when considering your emergency medicine job opportunities.*<br><br>Predictable start and end time each workday	radio (Matrix)<br>1 Very important<br>2 Important<br>3 Of little importance<br>4 Not important at all<br>5 Not applicable
152	imp_workday_length<br><br>Show the field ONLY if: [job_offer] = '1'	Length of each workday	radio (Matrix)<br>1 Very important<br>2 Important<br>3 Of little importance<br>4 Not important at all<br>5 Not applicable
153	imp_night_call<br><br>Show the field ONLY if: [job_offer] = '1'	Frequency of overnight shifts	radio (Matrix)<br>1 Very important<br>2 Important<br>3 Of little importance<br>4 Not important at all

			5 Not applicable
154	imp_weekend_duties<br>Show the field ONLY if:<br>[job_offer] = '1'	Frequency of weekend duties	radio (Matrix)<br>1 Very important<br>2 Important<br>3 Of little importance<br>4 Not important at all<br>5 Not applicable
155	imp_desired_location<br>Show the field ONLY if:<br>[job_offer] = '1'	Job/practice in desired location	radio (Matrix)<br>1 Very important<br>2 Important<br>3 Of little importance<br>4 Not important at all<br>5 Not applicable
156	imp_practice_setting<br>Show the field ONLY if:<br>[job_offer] = '1'	Job/practice in desired practice setting (e.g., hospital, group practice, etc.)	radio (Matrix)<br>1 Very important<br>2 Important<br>3 Of little importance<br>4 Not important at all<br>5 Not applicable
157	imp_visa_reqs<br>Show the field ONLY if:<br>[job_offer] = '1'	Job/practice meets visa status requirements	radio (Matrix)<br>1 Very important<br>2 Important<br>3 Of little importance<br>4 Not important at all<br>5 Not applicable
158	imp_salary_comp<br>Show the field ONLY if:<br>[job_offer] = '1'	Salary/compensation	radio (Matrix)<br>1 Very important<br>2 Important<br>3 Of little importance<br>4 Not important at all<br>5 Not applicable
159	imp_part_time<br>Show the field ONLY if:<br>[job_offer] = '1'	Availability of part-time position	radio (Matrix)<br>1 Very important<br>2 Important<br>3 Of little importance<br>4 Not important at all<br>5 Not applicable

160	imp_parental_leave<br><br>Show the field ONLY if:<br>[job_offer] = '1'	Parental leave policy	radio (Matrix)<br>1 Very important<br>2 Important<br>3 Of little importance<br>4 Not important at all<br>5 Not applicable
161	imp_partnshp<br><br>Show the field ONLY if:<br>[job_offer] = '1'	Partnership Opportunity	radio (Matrix)<br>1 Very important<br>2 Important<br>3 Of little importance<br>4 Not important at all<br>5 Not applicable
162	imp_cost_living<br><br>Show the field ONLY if:<br>[job_offer] = '1'	Cost of living	radio (Matrix)<br>1 Very important<br>2 Important<br>3 Of little importance<br>4 Not important at all<br>5 Not applicable
163	imp_taxes<br><br>Show the field ONLY if:<br>[job_offer] = '1'	Staying in the same city/region as my EM training program	radio (Matrix)<br>1 Very important<br>2 Important<br>3 Of little importance<br>4 Not important at all<br>5 Not applicable
164	imp_employment_spouse<br><br>Show the field ONLY if:<br>[job_offer] = '1'	Employment opportunities for spouse/partner	radio (Matrix)<br>1 Very important<br>2 Important<br>3 Of little importance<br>4 Not important at all<br>5 Not applicable
165	imp_proximity_family<br><br>Show the field ONLY if:<br>[job_offer] = '1'	Proximity to family	radio (Matrix)<br>1 Very important<br>2 Important<br>3 Of little importance<br>4 Not important at all<br>5 Not applicable
166	imp_climate	Climate/weather	radio (Matrix)

	Show the field ONLY if: [job_offer] = '1'		1 Very important<br>2 Important<br>3 Of little importance<br>4 Not important at all<br>5 Not applicable
167	imp_hosp_type<br><br>Show the field ONLY if: [job_offer] = '1'	Type of hospital (i.e. trauma level)	radio (Matrix)<br>1 Very important<br>2 Important<br>3 Of little importance<br>4 Not important at all<br>5 Not applicable
168	imp_patient_pop<br><br>Show the field ONLY if: [job_offer] = '1'	Patient population to be served	radio (Matrix)<br>1 Very important<br>2 Important<br>3 Of little importance<br>4 Not important at all<br>5 Not applicable
169	imp_commy<br><br>Show the field ONLY if: [job_offer] = '1'	Type of community (i.e. urban/rural/suburban)	radio (Matrix)<br>1 Very important<br>2 Important<br>3 Of little importance<br>4 Not important at all<br>5 Not applicable
170	imp_org_type<br><br>Show the field ONLY if: [job_offer] = '1'	Organizational structure of practice (i.e. large national organization, regional group, individual hospital, etc)	radio (Matrix)<br>1 Very important<br>2 Important<br>3 Of little importance<br>4 Not important at all<br>5 Not applicable
171	imp_research<br><br>Show the field ONLY if: [job_offer] = '1'	Opportunities for research	radio (Matrix)<br>1 Very important<br>2 Important<br>3 Of little importance<br>4 Not important at all<br>5 Not applicable
172	imp_teach<br><br>Show the field	Opportunities to teach	radio (Matrix)<br>1 Very important

	ONLY if: [job_offer] = '1'		2 Important<br>3 Of little importance<br>4 Not important at all<br>5 Not applicable
173	imp_supp_env<br><br>Show the field ONLY if: [job_offer] = '1'	A supportive academic environment	radio (Matrix)<br>1 Very important<br>2 Important<br>3 Of little importance<br>4 Not important at all<br>5 Not applicable
174	imp_mentors<br><br>Show the field ONLY if: [job_offer] = '1'	Availability of potential mentors	radio (Matrix)<br>1 Very important<br>2 Important<br>3 Of little importance<br>4 Not important at all<br>5 Not applicable
175	imp_np_pa<br><br>Show the field ONLY if: [job_offer] = '1'	Use of NPs, PAs and other clinical staff	radio (Matrix)<br>1 Very important<br>2 Important<br>3 Of little importance<br>4 Not important at all<br>5 Not applicable
176	imp_other<br><br>Show the field ONLY if: [job_offer] = '1'	Other factor(s)	radio (Matrix)<br>1 Very important<br>2 Important<br>3 Of little importance<br>4 Not important at all<br>5 Not applicable
177	imp_job_factors_other<br><br>Show the field ONLY if: [imp_other]='1' or [imp_other]='2' or [imp_other]='3' or [imp_other]='4' or [imp_other]='5'	Please describe the other factors you rated when considering EM opportunities.	text<br>Custom alignment: LV
178	em_jobs_50_miles	Section Header: *JOB MARKET PERCEPTIONS*<br><br>What is your overall assessment of	radio<br>1 Many jobs

	Show the field ONLY if: [job_search]='1'	Emergency Medicine positions within 50 miles of your training site?	2 Some jobs<br>3 Few jobs<br>4 Very few jobs<br>5 No jobs<br>6 I don't know<br><br>Custom alignment: LV
179	em_jobs_national<br><br>Show the field ONLY if: [job_search]='1'	What is your overall assessment of Emergency Medicine opportunities nationally?	radio<br>1 Many jobs<br>2 Some jobs<br>3 Few jobs<br>4 Very few jobs<br>5 No jobs<br>6 I don't know<br><br>Custom alignment: LV
180	recommend_em	Would you recommend Emergency Medicine to current medical students?	yesno<br>1 Yes<br>0 No<br><br>Custom alignment: LV
181	recommend_em_yes_explain<br><br>Show the field ONLY if: [recommend_em] = '1'	Please explain.	notes<br>Custom alignment: LV
182	recommend_em_no_explain<br><br>Show the field ONLY if: [recommend_em] = '0'	Please explain.	notes<br>Custom alignment: LV
183	income_var<br><br>Show the field ONLY if: [job_offer]='1'	How much salary variability (in terms of total annual income) was there across different job offers you received?	radio<br>1 Very little or none<br>2 Quite a bit<br>3 A great deal<br>4 Only got one offer so can't say<br><br>Custom alignment: LV
184	income_var_reason	What was the primary reason for this variability?	radio<br>1 Location

	Show the field ONLY if: [income_var]='2' or [income_var]='3'		2 Care setting (urgent care v. ED vs. academic center, etc)<br>3 Expected patient volume<br>4 Independent contractor status v. employee with benefits<br>5 Other<br>6 I don't know<br><br>Custom alignment: LV
185	income_var_reason_other<br><br>Show the field ONLY if: [income_var_reason]='5'	Please describe the other reason for salary variability.	text<br>Custom alignment: LV
186	when_decide_em	Section Header: *EDUCATIONAL EXPERIENCES AND PERCEPTIONS*<br><br>When did you decide you wanted to pursue Emergency Medicine as a specialty?	radio<br>1 Before medical school<br>2 During medical school<br>3 After practicing/training in a different specialty<br><br>Custom alignment: LV
187	work_with_nps_training	During your EM training, did you work with NPs in the emergency department?	yesno<br>1 Yes<br>0 No<br><br>Custom alignment: LV
188	work_with_pas_training	During your EM training, did you work with PAs in the emergency department?	yesno<br>1 Yes<br>0 No<br><br>Custom alignment: LV
189	satis_residency	Were you satisfied with your EM residency experience?	yesno<br>1 Yes<br>0 No<br><br>Custom alignment: LV
190	satis_residency_rsn<br><br>Show the field ONLY if: [satis_residency]='0'	Why were you not satisfied with your EM residency experience?	text<br>Custom alignment: LV

191	addtnl_comments	Do you have any additional comments regarding the specialty of emergency medicine you would like to share with the leadership of emergency medicine?	yesno<br>1 Yes<br>0 No<br><br>Custom alignment: LV
192	addtnl_comments_desc<br><br>Show the field ONLY if: [addtnl_comments]='1'	Please provide your additional comments here.	text<br>Custom alignment: LV
193	emergency_medicine_residents_survey_complete	Section Header: *Form Status*<br><br>Complete?	dropdown<br>0 Incomplete<br>1 Unverified<br>2 Complete
